# Association of everyday discrimination with health outcomes among Asian and non-Asian US older adults before and during the COVID-19 pandemic

**DOI:** 10.3389/fpubh.2022.953155

**Published:** 2022-10-19

**Authors:** Lulu Zhang, Mario Cruz-Gonzalez, Ziqiang Lin, Xinyi Ouyang, Fengnuan Zhao, Margarita Alegría

**Affiliations:** ^1^Disparities Research Unit, Department of Medicine, Massachusetts General Hospital, Boston, MA, United States; ^2^Department of Medicine, Harvard Medical School, Boston, MA, United States; ^3^Department of Psychiatry, Harvard Medical School, Boston, MA, United States

**Keywords:** everyday discrimination, depression and anxiety symptoms, level of functioning, sleep quality, Asian and non-Asian older adults, COVID-19

## Abstract

Increases in anti-Asian COVID-19 related discriminatory behaviors have been observed, many of which targeted older adults. Studies demonstrate that racial discrimination is associated with worse health outcomes, including anxiety, depression, and sleep difficulties. No previous studies have examined the impact of day-to-day experiences of discrimination before and during COVID-19 on both Asian and non-Asian older adults within the same sample. We examined whether everyday discrimination was associated with increased anxiety and depression symptoms, decreased levels of functioning, and increased sleep difficulties among Asian and non-Asian US older adults before and during the pandemic. Data came from the Positive Minds-Strong Bodies randomized clinical trial, an evidence-based mental health and disability prevention intervention for racially and ethnically diverse older adults with elevated depression or anxiety symptoms and minor to moderate disability. We conducted secondary data analyses in a cohort of 165 older adults (56 Asian and 109 non-Asian) assessed before COVID-19 (May 2015-May 2018) and during COVID-19 (March 2021-July 2022). Regression models examined whether everyday discrimination impacted health outcomes differently before and during COVID-19, and whether this effect was stronger among Asian compared to non-Asian older adults. Non-Asian older adults reported the same levels of everyday discrimination before and during COVID-19. Consistent with literature suggesting that social distancing has inadvertently kept US Asian populations from experiencing discrimination, Asian older adults reported marginally lower levels of everyday discrimination during the pandemic compared with pre-pandemic. We found that everyday discrimination was not associated with health outcomes before COVID-19. In contrast, during the pandemic, everyday discrimination was associated with worse anxiety and depression symptoms and worse levels of functioning, although only the impact on depression was significantly stronger compared with before the pandemic. This negative impact of everyday discrimination on health outcomes during the pandemic appeared to affect both Asian and non-Asian older adults similarly. Social support and social cohesion buffered against the negative effect of everyday discrimination on depression and level of functioning during the pandemic. Results suggest that public health interventions aimed at reducing everyday discrimination and emphasizing social support and cohesion can potentially improve health outcomes for all US older adult populations.

**Clinical trial registration:**

www.ClinicalTrials.gov; identifier: NCT02317432.

## Introduction

Due to misinformation about the novel coronavirus (e.g., referring to it as the “China virus” or “Kung Flu”), anti-Asian racism and xenophobic attacks against Asian Americans have significantly increased since the beginning of the COVID-19 pandemic (1–5). From March 19, 2020 to March 31, 2022, the data tracking system developed by the Stop Asian American and Pacific Islander (AAPI) Hate coalition reported a total of 11,467 hate incidents against AAPI persons (6). Since racial discrimination is a well-established risk factor of poor health outcomes (7), including depression and anxiety (8), posttraumatic stress (9), and sleep disruptions (10), several recent studies have examined the effect of anti-Asian COVID-19 related discrimination on Asian Americans' health. These studies have confirmed that racial discrimination during the COVID-19 pandemic is associated with worse depression, anxiety, and posttraumatic stress disorder symptoms, physical symptoms, and sleep quality (1–4).

This prior evidence on the association between anti-Asian COVID-19 related discrimination and health outcomes has been limited by two factors. The first one relates to the use of samples that include Asian populations only. The one exception is a prior study showing that both Asian and non-Asian US young adults had negative affective reactions to anti-Asian COVID-19 related discrimination (3). Yet, other minority populations have reported increased discrimination because of their race or ethnicity amid the COVID-19 pandemic. A recent study by the Pew Research Center showed that, compared to White Americans, Asian and Black Americans reported that it was more common for people to express racist views toward them since the coronavirus outbreak and that they feared someone might threaten or physically attack them because of their race (11). Asian, Black, and Latinx Americans were also more likely to report that people have acted as if they were uncomfortable around them and that they had been the subject of slurs or jokes (11). In addition, the relationship between discrimination and health outcomes has been found to differ by racial and ethnic groups. A recent meta-analysis, for example, showed that the association between racial discrimination and negative mental health is stronger for Asian and Latinx Americans compared with African Americans (7). However, there is limited research examining whether everyday experiences of discrimination during COVID-19 had a stronger impact on Asian than non-Asian US adults.

The second limitation relates to a lack of pre-pandemic baseline data against which to measure changes in discrimination within individuals. Although recent studies have confirmed that racial discrimination during COVID-19 is associated with poorer mental and physical health, in the absence of pre-pandemic data these studies implicitly assume that lower levels of discrimination would have been reported had the pandemic not happened. However, the pandemic has imposed some restrictions that could have led to decreased opportunities to experience discrimination. After the World Health Organization declared the COVID-19 outbreak a global pandemic on March of 2020, many US states enacted stay-at-home orders to prevent the spread of COVID-19. Whereas, social isolation itself can negatively affect mental and physical health (12), a recent study suggested that isolation due to stay-at-home orders could have served as a protective factor against experiencing discrimination for some Asian adults living in the US (4). In the same study, Asian and Asian Americans who reported not experiencing discrimination during COVID-19 attributed it to the fact that they lived in predominantly Asian communities (4). Thus, studies that include pre-pandemic data on racial discrimination are needed to reach conclusive evidence regarding its impact during COVID-19.

In addition, there is limited research on the association between racial discrimination and health outcomes during the pandemic among racial and ethnic minority older adults. Studying the effect of discrimination amid COVID-19 among these minoritized groups is relevant for at least four reasons. First, a recent report by the Stop AAPI Hate coalition showed that between March 2020 and December 2021, 7.6% of hate incidents reported by AAPI persons targeted older adults aged 60 years and above (13). These data also revealed that Asian American older adults who experienced hate incidents reported increased fear, stress, and anxiety during the pandemic than Asian American older adults overall (13). Second, although racial discrimination can impact mental and physical health outcomes across a range of populations, recent work has increasingly emphasized the potential compounding effect that ageism and racism can have on older adults (14–16). According to a 2021 editorial in the *Lancet Health Longevity* (14), ageism, independent of race, has become entrenched in health-care systems. Indeed, studies have shown that older adults receive inadequate care due to stereotypes, prejudice, and discrimination (16). Thus, by definition, the cumulative stress of racial discrimination can result in the most severe deterioration of mental and physical health in older adults. Third, because of high rates of morbidity and mortality, the pandemic has placed a disproportionate load on racial and ethnic minority older adults. From the beginning of the pandemic, being of older age became the strongest single determinant of all-cause mortality from COVID-19 (17). Research on the initial health effects of COVID-19 also demonstrates that US Asian, Black, and Latinx older adults are all at increased risk of death from COVID-19 compared to their White counterparts (18, 19). Lastly, also due to high rates of morbidity and mortality, older adults have been the most affected by increased social isolation because of stay-at-home restrictions (20). Increased social isolation has been found to be associated with decreased life satisfaction, higher levels of depression, and lower levels of psychological wellbeing (21–24), including during the COVID-19 pandemic (25). Further, widespread stay-at-home and social distancing measures during the pandemic may have made maintaining social support more challenging. Perceived discrimination can lead to increased psychological distress especially when individuals have limited access to resources for coping (26, 27), and social support has been highlighted as a common copying resource that can alleviate the effects of discrimination (28). Prior research has found consistent evidence that social support, including support from family and friends as well as neighborhood cohesion, is a protective factor that buffers against the negative effects of discrimination on health (29–31).

Using a cohort of 165 older adults who were assessed before and during COVID-19 in the context of a randomized clinical trial (RCT), the present study had three aims. Our first aim was to investigate changes in self-reported everyday discrimination and mental and physical health (depression and anxiety symptoms, level of functioning, and sleep difficulties) within Asian and non-Asian older adults before and during COVID-19. Our second aim sought to quantify the association between self-reported everyday discrimination and mental and physical health before and during COVID-19, and to test whether these associations were stronger among Asian older adults compared with non-Asian older adults. In alignment with prior research, our last goal was to examine whether social support and social cohesion were possible moderators altering the association between discrimination and health outcomes of older adults.

## Methods

### Study design and setting

This is a secondary analysis using data from the Positive Minds-Strong Bodies (PMSB) randomized clinical trial, a disability preventive intervention aimed to improve mental health and physical functioning of racial and ethnic minority older adults with mild to severe depression or anxiety symptoms and minor to moderate disability (32). Research assistants blinded to intervention condition conducted screening, baseline, and follow-up assessments between May 2015 and March 2019. Participants were recruited from community-based organizations (CBOs) and community clinics serving low-income older adults in Massachusetts, New York, Florida, and Puerto Rico. After assessing their capacity to consent, potential study participants completed a screening assessment to determine eligibility. Eligible participants who agreed to participate completed a baseline assessment and then were randomized to either PMSB or enhanced usual care (EUC). Follow-up assessments were conducted at 2-, 6-, and 12-months post-baseline.

As part of a follow-up study aimed to assess the impact of the COVID-19 pandemic among older adults previously enrolled in PMSB, research assistants re-contacted study participants between March 2021 and July 2022 and invited them to participate in a COVID-19 follow-up assessment. Self-reported measures of mental and physical health were included in all assessments (i.e., baseline, 2-, 6-, and 12-month follow-up, and COVID-19 follow-up). However, self-reported everyday discrimination was included in the baseline and COVID-19 follow-up assessments only. Thus, the present study used the baseline assessment, conducted between May 2015 and May 2018, as the only pre-pandemic data. The COVID-19 follow-up assessment, collected between March 2, 2021, and July 18, 2022, was used as the during-pandemic data. Study participants completed the COVID-19 follow-up assessment ~2.9–6.4 years post-baseline. In the initial RCT, study procedures were approved by the Institutional Review Boards of Massachusetts General Hospital/Partners HealthCare and New York University, with ceded reviews for partnering CBOs conducting human subjects' research. The Institutional Review Boards for Massachusetts General Hospital and New York University approved the ongoing COVID-19 follow-up assessment. All participants provided informed consent.

### Participants

In the initial RCT, 1,057 potential participants were screened at the participating CBOs and community clinics to assess eligibility. Eligible participants were 60 years old and above, spoke either English, Spanish, Mandarin, or Cantonese, and presented mild to severe depression or anxiety symptoms and minor to moderate disability. Mild to severe depression symptoms were defined as scoring five and above on either the Patient Health Questionnaire-9 (PHQ-9) (33) or the Geriatric Depression Scale-15 (GDS-15) (34). Mild to severe anxiety symptoms were defined as scoring five and above on the Generalized Anxiety Disorder-7 (GAD-7) (35). Minor to moderate disability was defined as scoring between three and 11 on the Short Physical Performance Battery (SPPB) (36). Exclusion criteria included disclosure of substance use disorders, having received mental health treatment in the previous 3 months or having an appointment within the following month, lacking capacity to consent, being homebound, having a neuromusculoskeletal impairment, or not receiving medical clearance for exercise from a physician. Participants disclosing serious suicide plans or suicide attempts on the Paykel Suicide Risk Questionnaire (37) were referred to emergency health services and re-screened after 30 days. These exclusion criteria were applied because of the following reasons. The PMSB is a combined psychosocial and exercise training intervention. The psychosocial intervention was offered by community health workers (CHWs) who were trained approximately 80 h in providing resources for better coping with depression, anxiety, and stress. Thus, people disclosing substance use disorders were excluded because that would require expertise outside the one CHWs were trained for. We excluded people receiving mental health services because the emphasis was on servicing those with no available treatments, given the shortage of mental health resources. However, people who were seeing psychiatrists for psychotropic medication were included in the study. People lacking capacity to consent were not included because of ethical reasons. Finally, people that were homebound, had a neuromusculoskeletal impairment, or whose physicians did not give them medical clearance for exercise were not eligible because the RCT also tested the combined effect of an exercise training intervention.

**Figure 1:**
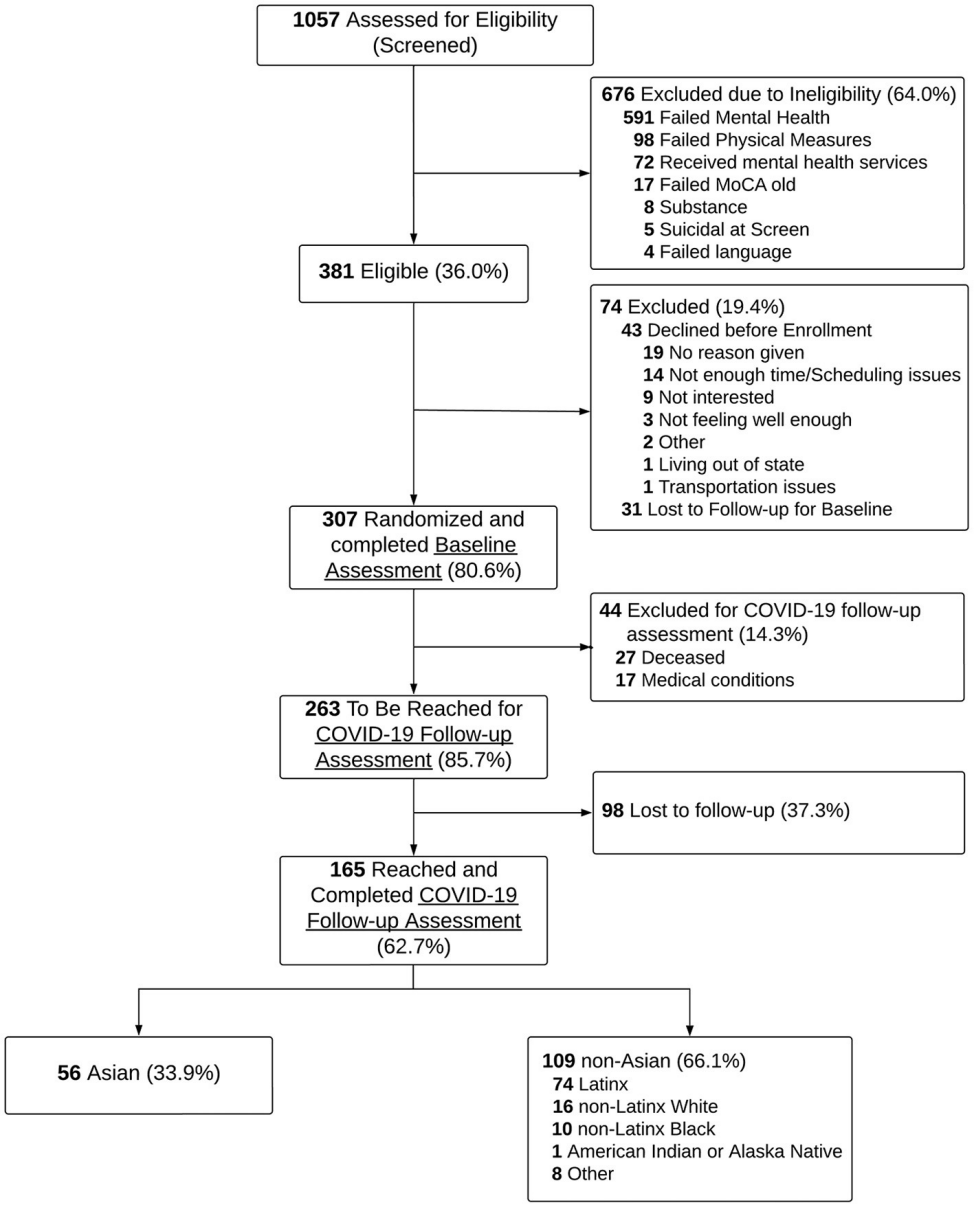
Consort diagram of the study design.

In total, 381 (36.0%) screened participants were eligible, of which 307 (80.6%) agreed to participate and were randomized to either PMSB or EUC conditions. In the COVID-19 follow-up study, we attempted to re-contact 302 of the 307 previously enrolled participants, as five of them were identified as deceased according to study records. An additional 20 participants have been identified as deceased from the National Death Index, and two more during COVID-19 follow-up data collection. Further, 17 participants were re-contacted but unable to participate due to medical conditions (e.g., cognitively impaired or severely ill). Among the remaining 263 participants, 165 (62.7%) were successfully reached and completed the COVID-19 follow-up assessment *via* phone interviews (56 Asian, 74 Latinx, 16 non-Latinx White, 10 non-Latinx Black, 1 American Indian or Alaska Native, and 8 of other-race). Because of small sample size in some racial and ethnic groups, all 109 non-Asian older adults were analyzed together (see Consort Diagram in Figure 1).

### Interventions

The PMSB is a combined psychosocial and exercise training intervention. The psychosocial intervention, which was delivered within 6 months, included ten one-h individual sessions focused on psychoeducation, mindfulness, cognitive restructuring, noticing and overcoming unhelpful thoughts, and creating a self-care plan. The exercise training intervention, delivered within 12–14 weeks and concurrently with the psychosocial intervention, included 36 group sessions of physical exercise focused on enhancing functioning and preventing physical disability. As described in Alegría et al. (32), the intervention was found to improve self-reported depression and anxiety symptoms, and self-reported and objectively measured physical functioning at 6-month follow-up (32). In addition, improvements in self-reported outcomes were maintained 6 months post-intervention at 12-month follow-up (32).

### Measures

#### Outcomes: Depression and anxiety symptoms, level of functioning, and sleep difficulties at baseline (pre-pandemic) and COVID-19 follow-up

Depression symptoms were assessed using the GDS-15, a 15-item self-reported measure used to screen, diagnose, and evaluate depression in older adults (34). The measure can be easily used by physically ill and mildly to moderately cognitively impaired older adults who have short attention spans and/or feel easily fatigued. The scale has a 92% sensitivity and a 89% specificity when evaluated against diagnostic criteria for depression (38). Respondents are asked to endorse yes or no questions about how they felt the last week. Of the 15 items, 10 indicate the presence of depression when answered positively, and five indicate the presence of depression when answered negatively. Total scores are calculated by summing all items (range: 0–15), and higher scores represent worse symptoms. Internal consistency (Cronbach's α) was adequate both at baseline and COVID-19 follow-up within Asian and non-Asian older adults (Asian α: 0.74 and 0.83 pre- and during-pandemic; non-Asian α: 0.72 and 0.77 pre- and during-pandemic). Anxiety symptoms were assessed using the (GAD-7), a 7-item self-reported measure to identify probable cases of Generalized Anxiety Disorder (GAD), which is characterized by excessive worry and persistent anxiety. The instrument has been widely used to screen for GAD and to monitor changes in anxiety symptoms over time. Respondents are asked how often have they been bothered by each of the core GAD symptoms in the past 2 weeks (e.g., feeling nervous, anxious, or on edge; worrying too much; becoming easily annoyed) (35). Responses are rated on a 4-point scale (0 = *not at all* and 3 = *nearly every day*). Total scores are calculated summing all items (range: 0 to 21; Asian α: 0.86 and 0.92 pre- and during-pandemic; non-Asian α: 0.79 and 0.88 pre- and during-pandemic), with higher scores representing worse symptoms.

Level of functioning was assessed using the Function Component of the Late-life Functioning and Disability Instrument (Late-life FDI), a 32-item self-reported measure for older adults that assess difficulties performing daily physical activities without help from others or assisted devices (39). The instrument has been widely used among community-dwelling older adults, with tested reliability and validity (40). Responses are rated on a 5-point scale (1 = *cannot do* and 5 = *none*). Total scores are calculated summing all items (range: 32 to 160; Asian α: 0.95 and 0.95 pre- and during-pandemic; non-Asian α: 0.96 and 0.97 pre- and during-pandemic), and higher scores represent greater levels of physical functioning. Sleep difficulties were assessed using five items about the following past-month self-reported difficulties with sleep: Falling asleep, staying asleep, waking up too early, frequent awakening during the night, and sleeping during the day. Responses are rated on a 4-point scale (0 = *never* and 3 = three *or more times a week*). Total scores were calculated summing all items (range: 0–15; Asian α: 0.69 and 0.67 pre- and during-pandemic; non-Asian α: 0.66 and 0.81 pre- and during-pandemic), and higher scores represent lower sleep quality.

#### Exposure: Self-reported everyday discrimination at baseline (pre-pandemic) and COVID-19 follow-up

The 9-item Everyday Discrimination Scale (EDS) was used to assess the frequency of day-to-day experiences with unfair treatment that are chronic or episodic but generally minor (e.g., “you are treated with less courtesy than other people,” “people act as if they are afraid of you,” or “you are called names or insulted”) (41). Because the items within the EDS are framed rather generically, the scale has been used to measure everyday experiences of discrimination for a variety of racial and ethnic groups (including persons who self-identified as non-Latinx White) and has been found to have adequate psychometric properties. Prior studies examining racial and ethnic differences in responses to the EDS have also found that the measure can potentially be used across racial and ethnic groups as originally intended (42–45). Responses are rated on a 6-point scale (0 = *never* and 5 = *almost every day*). Total scores were calculated summing all items (range: 0–45; Asian α: 0.91 and 0.82 pre- and during-pandemic; non-Asian α: 0.89 and 0.83 pre- and during-pandemic), and higher scores represent higher levels of perceived discrimination. Because on average non-Latinx White populations tend to report lower levels of discrimination compared to other racial and ethnic groups, we conducted sensitivity analyses that excluded non-Latinx White older adults from our sample. Since our main findings were not affected by removing non-Latinx White participants, we chose not to exclude them from our analyses.

#### Moderators: Social support and social cohesion at baseline (pre-pandemic) and COVID-19 follow-up

Social support was assessed using 10 items about the quantity and quality of social support (46). Four items assessed emotional social support by asking about the frequency of discussing problems with family, friends, and spouse/partner (0 = *never* and 3 = *always*). Three items assessed instrumental social support by asking whether the respondent could depend on either relatives, neighbors/friends, or spouse/partner for help with practical things (0 = *no* and 3 = *yes*). One item assessed satisfaction with social support (0 = *very unsatisfied* and 3 = *very satisfied*) and the remaining two items asked about frequency of getting together with family and friends and frequency of being taken care of by family members (0 = *never* and 3 = *at least once a week*). Total scores are computed by averaging all items (range: 0 to 3; Asian α: 0.67 and 0.73 pre- and during-pandemic; non-Asian α: 0.58 and 0.52 pre- and during-pandemic), and higher scores indicate greater quantity and quality of social support. Social cohesion was assessed using the social cohesion and trust section of the Collective Efficacy Scale (47), a self-reported measure of how well-communities work together to make things happen. Respondents were asked how true each of the following statements were about their neighborhood: “People in this neighborhood can be trusted,” “People in this neighborhood generally get along with each other,” “I have neighbors who would help me if I had an emergency,” and “People in my neighborhood look out for each other.” Items are rated on a 4-point scale (0 = *not at all true* and 3 = *very true*). Total scores were computed as the sum of all items (range: 0–12; Asian α: 0.74 and 0.70 pre- and during-pandemic; non-Asian α: 0.75 and 0.81 pre- and during-pandemic), and higher scores represent greater neighborhood social cohesion.

#### Additional baseline (pre-pandemic) sociodemographic characteristics

We used baseline data to adjust for age, sex (male or female), education level (less than high school or high school and above), and intervention condition (PMSB or EUC). We also used baseline data to characterize participants in terms of household size, birthplace (foreign born or US born), and primary language (English, Spanish, Mandarin, or Cantonese). We noted that all Asian older adults in our sample were foreign born and reported their primary language as either Mandarin or Cantonese. Further, all non-Asian older adults reported their primary language as either English or Spanish. Thus, neither birthplace nor primary language were adjusted for because the effect of these two individual characteristics was indistinguishable from the effect of Asian race.

### Statistical analysis

We began by describing differences in the distribution of pre-pandemic baseline data between participants who completed and did not complete the COVID-19 follow-up assessment within Asian and non-Asian older adults. Examining these differences allowed us to assess whether, for example, older adults with higher levels of perceived discrimination or worse mental health symptoms were more likely to not have responded to the COVID-19 follow-up assessment. We then described changes in self-reported everyday discrimination and mental and physical health (depression and anxiety symptoms, level of functioning, and sleep difficulties) within Asian and non-Asian older adults before and during COVID-19. Afterwards, we examined the association between self-reported discrimination and mental and physical health outcomes before and during COVID-19 using linear regression models and tested whether these associations were stronger among Asian compared with non-Asian older adults. Mental and physical health outcomes were separately modeled as the dependent variable. Further, separate linear regression models were estimated using baseline pre-pandemic data and COVID-19 follow-up data. However, because self-reports before and during COVID-19 were likely correlated within individuals, regression models were estimated using a system of two linear equations, one using the baseline pre-pandemic data and another the COVID-19 follow-up data. These two regressions are related because the error term associated with the dependent variable may be correlated. By explicitly modeling this potential correlation, we could also test whether the effect of discrimination on health outcomes was stronger during COVID-19 compared with the pre-pandemic period. The system of equations representing this conceptualization was as follows:


$$\begin{array}{l} y_{i}^{0}=\beta_{1}^{0}+\beta_{2}^{0}Asian_{i}+\beta_{3}^{0}Discrimination_{i}^{0}+\beta_{4}^{0}\\ \;\left(Asian_{i}\times Discrimination_{i}^{0}\right)+\beta_{5}^{0}Social\;Support_{i}^{0}\\ \;+\beta_{6}^{0}Social\;Cohesion_{i}^{0}+\beta_{7}^{0}X_{i}+u_{i}^{0} \end{array}$$

$$\begin{array}{l} y_{i}^{1}=\beta_{1}^{1}+\beta_{2}^{1}Asian_{i}+\beta_{3}^{1}Discrimination_{i}^{1}+\beta_{4}^{1}\\ \;\left(Asian_{i}\times Discrimination_{i}^{1}\right)+\beta_{5}^{1}Social\;Support_{i}^{1}\\ \;+\beta_{6}^{1}Social\;Cohesion_{i}^{1}+\beta_{7}^{1}X_{i}+u_{i}^{1} \end{array}$$

where $y_{i}^{0}\;$represents an outcome variable for older adult *i* pre-pandemic and $y_{i}^{1}$ represents an outcome variable for older adult *i* during COVID-19. The superscript “0” is used to indicate a self-reported measure at the pre-pandemic baseline assessment, and the superscript “1” is used to indicate a self-reported measure at the COVID-19 follow-up. **X**_**i**_ is the vector of covariates that were adjusted for (i.e., age, sex, education level, and intervention condition). The error terms $u_{i}^{0}$ and $u_{i}^{1}$ are allowed to be correlated.

In the above system of equations, the parameter $\beta_{3}^{0}$ represents the effect of everyday discrimination pre-pandemic and the parameter $\beta_{4}^{0}$ tests whether this effect was stronger among Asian compared with non-Asian older adults. Analogously, the parameter $\beta_{3}^{1}$ represents the effect of everyday discrimination during COVID-19 and the parameter $\beta_{4}^{1}$ tests whether this effect was stronger among Asian compared with non-Asian older adults. Finally, we tested whether social support and social cohesion moderated the association between discrimination and health outcomes by adding two-way interactions to the above system of equations between self-reported discrimination and social support and cohesion. All analyses were performed in the Stata software version 15 (48).

## Results

### Descriptive characteristics of study sample

In Table 1, we present the pre-pandemic baseline distribution of all study variables for Asian and non-Asian older adults by whether they completed the COVID-19 follow-up assessment. Asian older adults who were re-contacted and completed the COVID-19 follow-up had pre-pandemic baseline GDS-15 and GAD-7 scores indicative of mild depression (34) and minimal anxiety (35), respectively. Their Late-Life FDI scores indicated moderate functional limitations (39). Most of these older adults were 75 years and older (64.3%) and the majority were females (78.6%). Three fourths reported having a high school degree and above. All were foreign born and reported their primary language as either Mandarin (57.1%) or Cantonese (42.9%). Except for education level and primary language, no significant differences in pre-pandemic baseline data were observed among Asian older adults who completed the COVID-19 follow-up compared with Asian older adults who did not. This result suggested that Asian older adults with worse health outcomes, higher levels of discrimination, and lower levels of social support were not less likely to complete the COVID-19 follow-up assessment.

**Table 1:** Distribution of pre-pandemic baseline variables among Asian and non-Asian older adults by whether they completed the COVID-19 follow-up assessment.

	**Asian (*****n*** = **102)**			**Non-Asian (*****n*** = **205)**^**a**^		
	**COVID-19 Follow-up**	**No COVID-19 Follow-up**	***P***	**COVID-19 Follow-up**	**No COVID-19 Follow-up**	***P***
**Pre-pandemic baseline variables**	**(*n* = 56)**	**(*n* = 46)**		**(*n* = 109)**	**(*n* = 96)**	
**Outcomes: self-reported health**						
Depression symptoms, GDS-15, mean (SD)	7.0 (3.3)	7.2 (3.2)	0.77	4.5 (3.0)	5.0 (3.1)	0.30
Anxiety symptoms, GAD-7, mean (SD)	4.3 (4.6)	5.3 (4.7)	0.27	6.3 (4.3)	6.9 (4.7)	0.39
Level of functioning, Late-Life FDI, mean (SD)	123.1 (22.3)	122.5 (27.4)	0.89	114.6 (26.8)	115.4 (26.1)	0.82
Sleep difficulties, mean (SD)	9.9 (4.0)	9.1 (3.9)	0.29	9.0 (3.6)	8.6 (3.7)	0.45
**Exposure: everyday discrimination, mean (SD)**	4.5 (7.4)	5.1 (6.4)	0.68	6.7 (8.1)	7.4 (9.2)	0.56
**Moderators: social support and social cohesion**						
Social support, mean (SD)	1.4 (0.6)	1.2 (0.6)	0.14	1.4 (0.5)	1.3 (0.6)	0.16
Social cohesion, mean (SD)	7.9 (1.9)	8.0 (2.3)	0.83	7.8 (2.8)	7.8 (2.9)	0.90
**Sociodemographic characteristics**						
**Age**, ***n*** **(%)**						
60–64	0 (0.0%)	2 (4.3%)	0.27	12 (11.0%)	7 (7.3%)	0.40
65–74	20 (35.7%)	14 (30.4%)		55 (50.5%)	44 (45.8%)	
75+	36 (64.3%)	30 (65.2%)		42 (38.5%)	45 (46.9%)	
**Gender**, ***n*** **(%)**						
Male	12 (21.4%)	11 (23.9%)	0.77	13 (11.9%)	23 (24.0%)	0.02
Female	44 (78.6%)	35 (76.1%)		96 (88.1%)	73 (76.0%)	
**Education**, ***n*** **(%)**						
Less the high school	14 (25.0%)	25 (54.3%)	0.002	36 (33.0%)	36 (37.5%)	0.50
High school or higher	42 (75.0%)	21 (45.7%)		73 (67.0%)	60 (62.5%)	
**Household size, mean (SD)**	1.8 (1.0)	1.9 (0.9)	0.64	2.1 (2.8)	1.9 (1.6)	0.50
**Birthplace**, ***n*** **(%)**						
Foreign born	56 (100.0%)	46 (100.0%)	NA	59 (55.1%)	49 (52.7%)	0.73
US born	0 (0.0%)	0 (0.0%)		48 (44.9%)	44 (47.3%)	
**Primary language**, ***n*** **(%)**						
English	0 (0.0%)	1 (2.2%)	0.02	34 (31.2%)	31 (32.3%)	0.31
Spanish	0 (0.0%)	0 (0.0%)		75 (68.8%)	63 (65.6%)	
Mandarin	32 (57.1%)	14 (30.4%)		0 (0.0%)	2 (2.1%)	
Cantonese	24 (42.9%)	31 (67.4%)		0 (0.0%)	0 (0.0%)	
**Intervention condition**, ***n*** **(%)**						
PMSB	31 (55.4%)	20 (43.5%)	0.23	46 (42.2%)	56 (58.3%)	0.01
EUC	25 (44.6%)	26 (56.5%)		63 (57.8%)	40 (41.7%)	

GDS-15, Geriatric Depression Scale-15; GAD-7, Generalized Anxiety Disorder-7; Late-life FDI, Late-Life Functioning and Disability Instrument; PMSB, Positive Minds-Strong Bodies; EUC, Enhanced Usual Care; NA, Not Applicable.

^a^The non-Asian groups includes older adults who self-identified as either non-Latinx White (n = 31), non-Latinx Black (n = 24), American Indian (n = 1), Latinx (n = 136), and other-race (n = 9).

As show in Table 1, non-Asian older adults who completed the COVID-19 follow-up were similar to Asian older adults in that they had baseline GDS-15, GAD-7, and Late-Life FDI scores indicative of mild depression (34), mild anxiety (35), and moderate functional limitations (39). Most of these non-Asian older adults were between 65 and 74 years old at baseline (50.5%), the majority were females (88.1%), and more than two thirds had a high school degree or more (67.0%). More than half (55.1%) were foreign born and they reported their primary language to be either English (31.2%) or Spanish (68.8%). Except for gender and intervention condition, we also found that non-Asian older adults with worse health outcomes, higher levels of discrimination, and lower levels of social support were equally likely to complete the COVID-19 follow-up assessment (i.e., no significant differences in pre-pandemic data were observed between non-Asian older adults who completed the COVID-19 follow-up and non-Asian older adults who did not).

### Changes in self-reported discrimination and health outcomes within individuals before and during COVID-19

**Figure 2:**
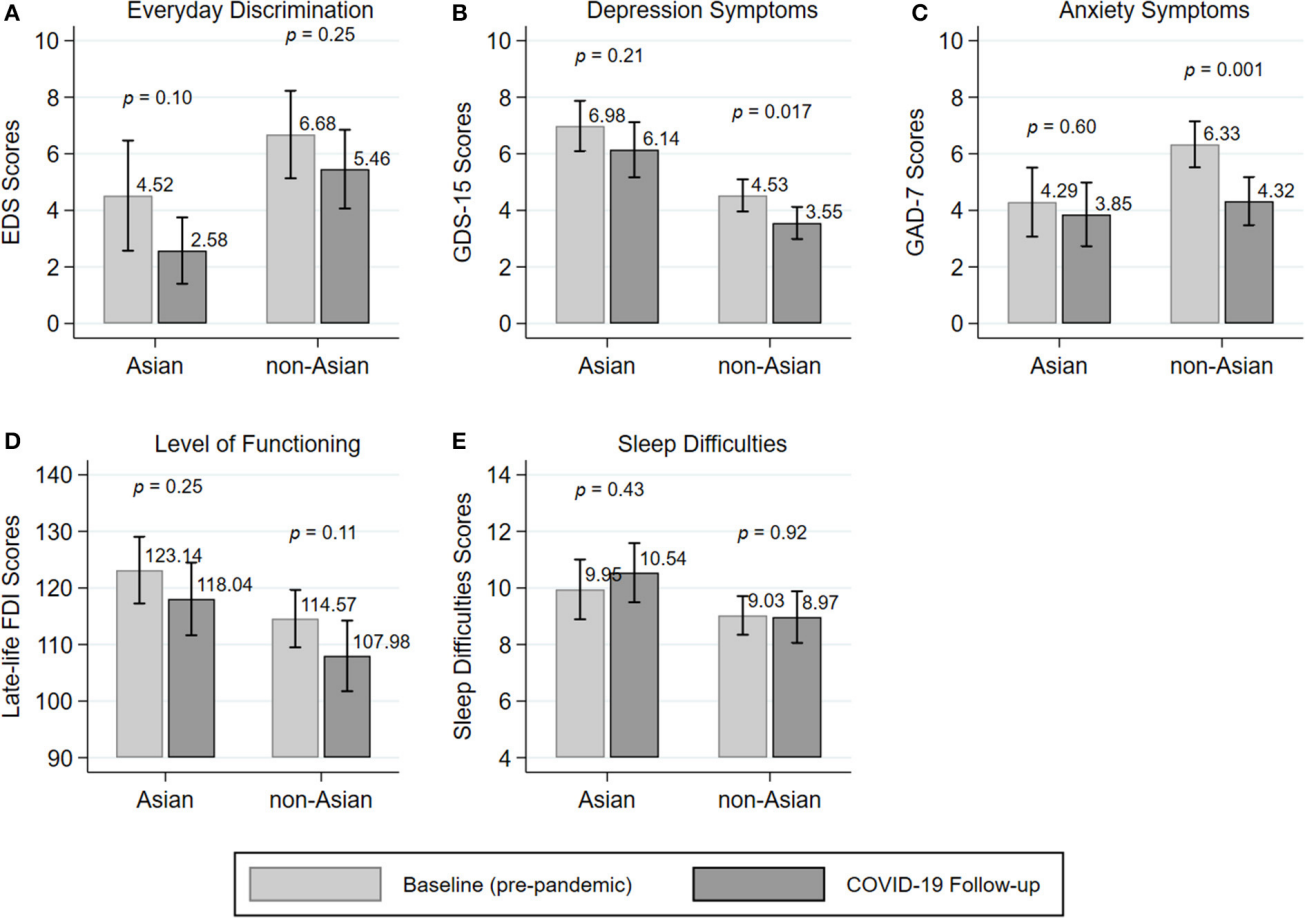
Changes in self-reported discrimination **(A)** and mental outcomes **(B,C)** and physical outcomes **(D,E)** within individuals before and during COVID-19.

In Figure 2, we present changes in everyday discrimination and health outcomes within Asian and non-Asian older adults before and during the COVID-19 pandemic. As shown in Figure 2A, before and during the pandemic Asian older adults reported lower levels of everyday discrimination compared with non-Asian older adults. Further, both Asian and non-Asian older adults reported less experiences of day-to-day unfair treatment during the pandemic compared with before the pandemic. However, lower levels of everyday discrimination during the pandemic were not significantly different compared with their pre-pandemic levels either among Asian older adults (4.52 before vs. 2.58 during COVID-19; *p* = 0.10) or among non-Asian older adults (6.68 before vs. 5.46 during COVID-19; *p* = 0.25).

Changes in depression and anxiety symptoms, level of functioning, and sleep difficulties within individuals before and during COVID-19 are presented in Figures 2B–E. Both, before and during the pandemic, Asian older adults reported higher depression symptoms and more sleep difficulties than non-Asian older adults. In addition, Asian older adults reported less anxiety symptoms and higher levels of functioning before and during COVID-19 than non-Asian older adults. Among Asian older adults, no change in either depression and anxiety symptoms, level of functioning, or sleep difficulties was observed from before the pandemic to during the pandemic. In contrast, among non-Asian older adults a significant decrease in depression and anxiety symptoms was observed from before to during the pandemic (depression symptoms: 4.53 before vs. 3.55 during COVID-19; *p* = 0.017; anxiety symptoms: 6.33 before vs. 4.32 during COVID-19; *p* = 0.001).

### Association between self-reported discrimination and health outcomes before and during COVID-19

Linear regression model estimates for the effect of everyday discrimination on health outcomes are presented in Table 2. As shown in Table 2, everyday discrimination was not associated with either worse depression and anxiety symptoms, lower levels of functioning, or increased sleep difficulties before the pandemic [column “Baseline (pre-pandemic)”]. In contrast, everyday discrimination was linked to higher depression (coefficient: 0.12; 95% Confidence Interval (CI): 0.04–0.20) and anxiety symptoms (coefficient: 0.21; 95% CI: 0.09–0.33) and lower levels of functioning (coefficient: −0.81; 95% CI: −1.47– −0.14) during the pandemic [column “COVID-19 follow-up”]. Only the impact of everyday discrimination on depression symptoms appeared to be significantly stronger during the pandemic compared with before the pandemic. To test whether the effect of self-reported discrimination was stronger among Asian compared with non-Asian older adults, the models from Table 2 included two-way interactions between Asian race and everyday discrimination (non-Asian race interacted with everyday discrimination served as the referent group). None of these two-way interactions were statistically significant, which suggested that a unit increase in everyday discrimination during COVID-19 had the same impact on Asian and non-Asian older adults' health outcomes.

**Table 2:** Association between self-reported discrimination and health outcomes before and during COVID-19.

	**A. Outcome variable: depression symptoms (GDS-15 scores)**		**B. Outcome variable: anxiety symptoms (GAD-7 scores)**	
	**Baseline (pre-pandemic)**	**COVID-19 follow-up**	**Baseline (pre-pandemic)**	**COVID-19 follow-up**
	**Coeff. [95% CI]**	**Coeff. [95% CI]**	**Coeff. [95% CI]**	**Coeff. [95% CI]**
**Race/ethnicity**				
Non-Asian (reference)				
Asian	2.19 [0.91, 3.48]*	2.57 [1.30, 3.84]*	−2.01 [−3.83, −0.19]*	0.10 [−1.65, 1.85]
**Everyday discrimination**	−0.05 [−0.12, 0.03]	0.12 [0.04, 0.20]*+	0.08 [−0.04, 0.19]	0.21 [0.09, 0.33]*
**Race/ethnicity x Everyday discrimination**				
Non-Asian (reference)				
Asian	0.06 [−0.06, 0.18]	0.06 [−0.13, 0.25]	0.04 [−0.15, 0.22]	0.09 [−0.20, 0.37]
**Social support**	−0.08 [−1.04, 0.88]	−0.63 [−1.47, 0.22]	−1.17 [−2.62, 0.28]	−0.23 [−1.48, 1.03]
**Social cohesion**	−0.08 [−0.27, 0.11]	−0.10 [−0.25, 0.04]	0.11 [−0.19, 0.40]	−0.12 [−0.34, 0.10]
**Adjustment variables** ^**a**^				
**Age**	−0.02 [−0.10, 0.06]	0.04 [−0.03, 0.12]	−0.08 [−0.19, 0.02]	0.01 [−0.09, 0.11]
**Gender**				
Male (reference)				
Female	0.01 [−1.39, 1.42]	0.12 [−1.26, 1.50]	−0.66 [−2.57, 1.26]	0.51 [−1.33, 2.35]
**Education**				
Less than high school (reference)				
High school or more	−0.32 [−1.48, 0.83]	−0.76 [−1.88, 0.36]	−0.22 [−1.80, 1.36]	−0.90 [−2.39, 0.59]
**Intervention condition**				
EUC (reference)				
PMSB	0.15 [−0.88, 1.18]	−0.77 [−1.78, 0.25]	0.69 [−0.71, 2.10]	0.28 [−1.07, 1.63]
	**C. Outcome variable: Level of functioning (Late-life FDI scores)**		**D. Outcome variable: Sleep difficulties**	
	**Baseline (pre-pandemic)**	**COVID-19 follow-up**	**Baseline (pre-pandemic)**	**COVID-19 follow-up**
	**Coeff. [95% CI]**	**Coeff. [95% CI]**	**Coeff. [95% CI]**	**Coeff. [95% CI]**
**Race/ethnicity**				
non-Asian (reference)				
Asian	8.80 [−0.96, 18.55]	11.02 [−0.07, 22.11]	1.80 [0.22, 3.39]*	1.60 [−0.14, 3.34]
**Everyday discrimination**	−0.27 [−0.79, 0.25]	−0.81 [−1.47, −0.14]*	0.05 [−0.04, 0.14]	0.04 [−0.07, 0.15]
**Race/ethnicity x Everyday discrimination**				
Non-Asian (reference)				
Asian	0.11 [−0.74, 0.95]	0.16 [−1.39, 1.70]	−0.09 [−0.23, 0.05]	−0.02 [−0.27, 0.24]
**Social support**	−0.17 [−6.87, 6.52]	3.05 [−3.78, 9.88]	0.26 [−0.87, 1.40]	−0.27 [−1.40, 0.85]
**Social cohesion**	1.45 [0.12, 2.77]*	1.13 [−0.07, 2.33]	0.05 [−0.18, 0.27]	−0.13 [−0.33, 0.07]
**Adjustment variables** ^**a**^				
**Age**	−0.98 [−1.61, −0.36]*	−1.24 [−1.95, −0.54]*	0.04 [−0.06, 0.14]	0.08 [−0.02, 0.19]
**Gender**				
Male (reference)				
Female	−8.94 [−19.96, 2.08]	−9.62 [−22.11, 2.86]	−0.11 [−1.87, 1.65]	−0.09 [−2.02, 1.83]
**Education**				
Less than high school (reference)				
High school or more	7.04 [−1.99, 16.07]	10.23 [0.12, 20.33]*	0.06 [−1.38, 1.50]	−0.02 [−1.58, 1.54]
**Intervention condition**				
EUC (reference)				
PMSB	−1.77 [−9.80, 6.26]	2.13 [−6.98, 11.25]	−0.22 [−1.50, 1.06]	−0.69 [−2.09, 0.72]

GDS-15, Geriatric Depression Scale-15; GAD-7, Generalized Anxiety Disorder-7; Late-life FDI, Late-life Functioning and Disability Instrument; Coeff., coefficient; PMSB, Positive Minds-Strong Bodies; EUC, Enhanced Usual Care.

^a^Adjustment variables of age, sex (male or female), education level (less than high school or high school and above), and intervention condition (PMSB or EUC) were all measured at baseline.

*p < 0.05; + Statistically significant difference at the 0.05 level compared with baseline (pre-pandemic).

None of the covariates that were adjusted for were significantly associated with the outcomes either before or during COVID-19 except for level of functioning. Results from Table 2 indicated that older age was associated with lower levels of functioning before and during COVID-19. In addition, during COVID-19, participants with a high school degree and above had higher levels of functioning compared to those who reported that they did not graduate from high school. Notably, although Asian older adults in the PMSB group were more likely to complete the COVID-19 follow-up assessment compared to non-Asian older adults, differences in intervention condition were also not significantly associated with the outcomes. This result suggested that everyday discrimination during COVID-19 having the same impact on Asian and non-Asian older adults was not related to differences in intervention condition.

### Social support and social cohesion as potential moderators altering the association between self-reported discrimination and health outcomes

To investigate whether social support and social cohesion moderated the negative impact of everyday discrimination on health outcomes during the pandemic, we estimated models that included two-way interactions between everyday discrimination and social support and cohesion. As shown in Table 3, neither social support nor social cohesion were associated with lower depression and anxiety symptoms or less sleep difficulties either before [column “Baseline (pre-pandemic)”] or during COVID-19 [column “COVID-19 follow-up”]. Although this result was also observed for level of functioning pre-pandemic, social cohesion was found to be associated with increased levels of functioning during the pandemic (b: 2.24; 95% CI: 0.72–3.76), and it also significantly buffered against the negative effect of discrimination (b: −0.23; 95% CI: −0.42– −0.03). While social support alone was not associated with lower depression symptoms in the COVID-19 follow-up data, the results from Table 3 indicated that it significantly buffered against the negative effect of discrimination on depression symptoms during the pandemic. No other two-way interaction was statistically significant.

**Table 3:** Social support and social cohesion as potential moderators altering the association between everyday discrimination and health outcomes.

	**E. Outcome variable: depression symptoms (GDS-15 scores)**		**F. Outcome variable: anxiety symptoms (GAD-7 scores)**	
	**Baseline (pre-pandemic)**	**COVID-19 follow-up**	**Baseline (pre-pandemic)**	**COVID-19 follow-up**
	**Coeff. [95% CI]**	**Coeff. [95% CI]**	**Coeff. [95% CI]**	**Coeff. [95% CI]**
**Race/ethnicity**				
Non-Asian (reference)				
Asian	2.50 [1.40, 3.60]*	2.77 [1.67, 3.86]*	−1.82 [−3.33, −0.31]*	0.32 [−1.13, 1.77]+
**Everyday discrimination**	−0.07 [−0.32, 0.18]	0.26 [0.08, 0.43]*+	0.26 [−0.12, 0.64]	0.50 [0.26, 0.75]*
**Social support**	0.13 [−1.01, 1.26]	−0.01 [−1.06, 1.04]	−1.05 [−2.78, 0.67]	0.42 [−1.11, 1.94]
**Social cohesion**	−0.12 [−0.34, 0.10]	−0.13 [−0.32, 0.06]	0.19 [−0.15, 0.53]	−0.04 [−0.32, 0.25]
**Everyday discrimination x Social support**	−0.03 [−0.17, 0.11]	−0.12 [−0.23, −0.004]*	−0.03 [−0.24, 0.18]	−0.13 [−0.30, 0.03]
**Everyday discrimination x Social cohesion**	0.01 [−0.01, 0.03]	0.00 [−0.02, 0.03]	−0.02 [−0.05, 0.02]	−0.02 [−0.06, 0.02]
**Adjustment variables** ^**a**^				
**Age**	−0.03 [−0.10, 0.05]	0.05 [−0.03, 0.12]	−0.09 [−0.20, 0.02]	0.02 [−0.08, 0.12]
**Gender**				
Male (reference)				
Female	0.02 [−1.38, 1.43]	0.22 [−1.16, 1.59]	−0.79 [−2.72, 1.14]	0.64 [−1.16, 2.45]
**Education**				
Less than high school (reference)				
High school or more	−0.37 [−1.52, 0.78]	−0.88 [−2.01, 0.25]	−0.24 [−1.82, 1.33]	−0.91 [−2.40, 0.58]
**Intervention condition**				
EUC (reference)				
PMSB	0.21 [−0.82, 1.24]	−0.74 [−1.73, 0.26]	0.62 [−0.79, 2.03]	0.34 [−0.96, 1.65]
	**G. Outcome variable: Level of functioning (Late-life FDI scores)**		**H. Outcome variable: Sleep difficulties**	
	**Baseline (pre-pandemic)**	**COVID-19 follow-up**	**Baseline (pre-pandemic)**	**COVID-19 follow-up**
	**Coeff. [95% CI]**	**Coeff. [95% CI]**	**Coeff. [95% CI]**	**Coeff. [95% CI]**
**Race/ethnicity**				
Non-Asian (reference)				
Asian	8.95 [0.38, 17.53]*	11.65 [1.90, 21.39]*	1.34 [−0.03, 2.71]	1.53 [0.01, 3.06]*
**Everyday discrimination**	0.53 [−1.20, 2.25]	0.12 [−1.29, 1.53]	−0.16 [−0.46, 0.13]	−0.01 [−0.24, 0.23]
**Social support**	2.36 [−5.51, 10.23]	−0.57 [−8.93, 7.78]	0.13 [−1.22, 1.47]	−0.26 [−1.66, 1.13]
**Social cohesion**	1.36 [−0.18, 2.90]	2.24 [0.72, 3.76]*	−0.04 [−0.31, 0.22]	−0.17 [−0.43, 0.09]
**Everyday discrimination x Social support**	−0.56 [−1.52, 0.41]	0.53 [−0.37, 1.43]	0.04 [−0.12, 0.21]	0.00 [−0.15, 0.15]
**Everyday discrimination x Social cohesion**	−0.02 [−0.18, 0.14]	−0.23 [−0.42, −0.03]*	0.02 [−0.01, 0.05]	0.01 [−0.03, 0.04]
**Adjustment variables** ^**a**^				
**Age**	−1.00 [−1.62, −0.38]*	−1.22 [−1.91, −0.52]*	0.04 [−0.06, 0.14]	0.08 [−0.02, 0.19]
**Gender**				
Male (reference)				
Female	−9.53 [−20.59, 1.52]	−10.22 [−22.56, 2.13]	0.05 [−1.71, 1.81]	−0.09 [−2.02, 1.84]
**Education**				
Less than high school (reference)				
High school or more	6.98 [−2.02, 15.98]	11.67 [1.57, 21.77]*	0.12 [−1.32, 1.56]	−0.05 [−1.62, 1.53]
**Intervention condition**				
EUC (reference)				
PMSB	−1.74 [−9.78, 6.30]	2.53 [−6.39, 11.45]	−0.13 [−1.41, 1.15]	−0.71 [−2.09, 0.68]

GDS-15, Geriatric Depression Scale-15; GAD-7, Generalized Anxiety Disorder-7; Late-life FDI, Late-life Functioning and Disability Instrument; Coeff., coefficient; PMSB, Positive Minds-Strong Bodies; EUC, Enhanced Usual Care.

^a^Adjustment variables of age, sex (male or female), education level (less than high school or high school and above), household size, and intervention condition (PMSB or EUC) were all measured at baseline.

*p < 0.05; + Statistically significant difference at the 0.05 level compared with baseline (pre-pandemic).

**Figure 3:**
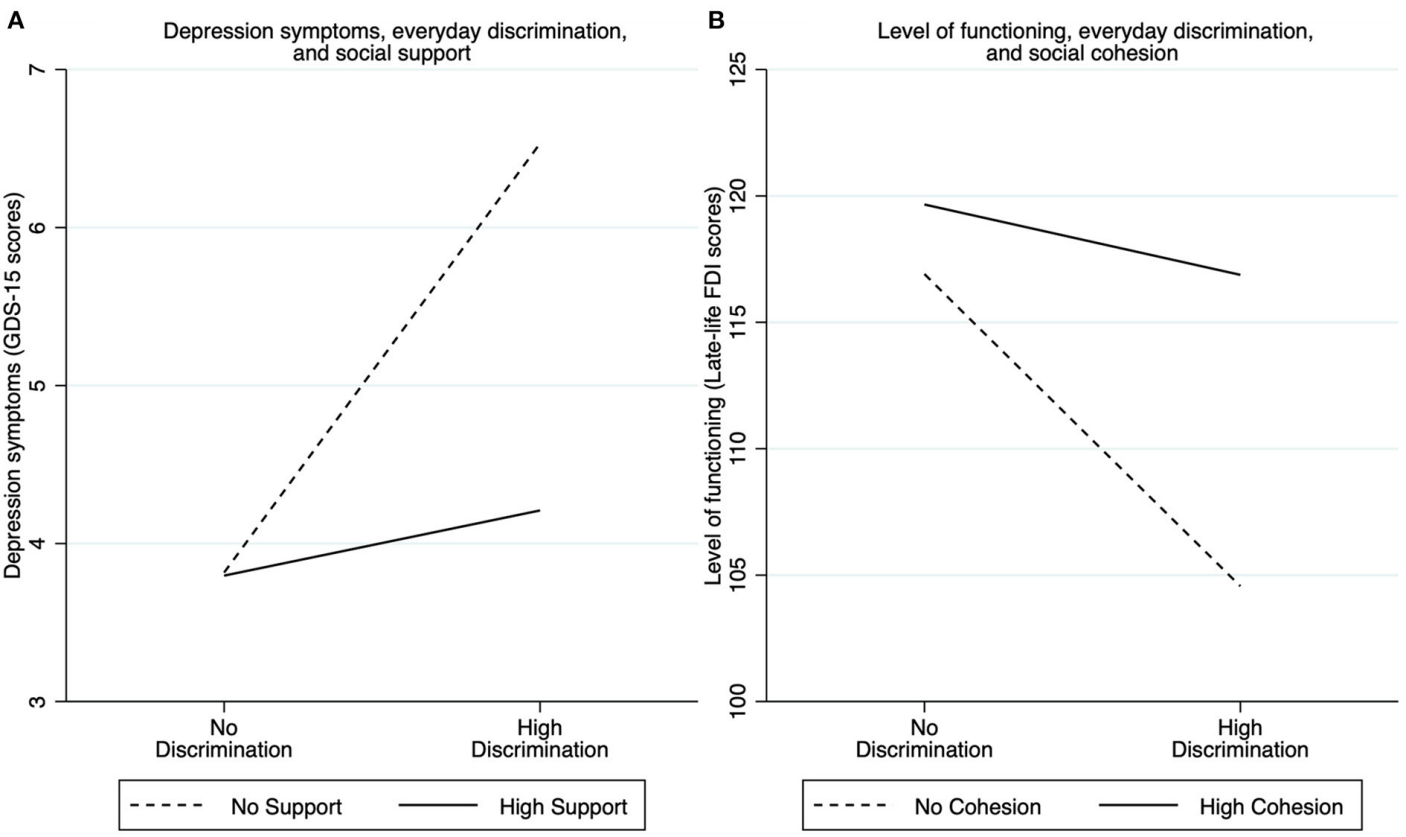
Simple slopes representing the moderating effect of social support altering the association between discrimination and depression symptoms **(A)** and the moderating effect of social cohesion altering the association between discrimination on level of functioning **(B)** during COVID-19. No discrimination was evaluated using an Everyday Discrimination Scale (EDS) score of 0, and high Discrimination as an EDS score at the mean + one-standard deviation. Similarly, no social support was evaluated using a score of 0, and high support as a score at the mean + one-standard deviation. No cohesion was evaluated using a score of 0, and high cohesion as a score at the mean + one-standard deviation.

In Figure 3, we depict simple slopes representing the moderating effect of social support altering the association between discrimination and depression symptoms (Figure 3A) and the moderating effect of social cohesion altering the association between discrimination on level of functioning (Figure 3B) during COVID-19. As shown in Figure 3A, increased discrimination (i.e., going from no discrimination to high levels of discrimination) led to worse depression symptoms (higher GDS-15 scores) for both participants without social support and participants with high levels of social support. However, increased discrimination worsened depression symptoms more for participants with no social support compared to participants with high levels of social support. Similarly, increased discrimination led to lower levels of functioning (lower Late-life FDI scores) for both participants without social cohesion and participants with high levels of social cohesion (Figure 3B). However, increased discrimination lowered level of functioning more among participants without social cohesion compared to participants with high social cohesion.

## Discussion

The present study is among the first to investigate within-person changes in self-reported discrimination before and during the COVID-19 pandemic among Asian and non-Asian US older adults. Contrary to our expectations, we found that Asian older adults reported fewer experiences of day-to-day unfair treatment (everyday discrimination) during the pandemic compared with before the pandemic (although this difference was not significant at the α = 0.05 level). Increased social isolation during COVID-19 may have resulted in decreased opportunities to experience discrimination. For example, some news reports have suggested that, to better protect themselves against exposure to COVID-19, many Asian older adults started isolating socially even before the stay-at-home orders were enforced (49). Recent studies also demonstrate that Asian older adults are at increased risk of social isolation from family and friends (50, 51). One study showed that during the pandemic people over the age of 50 had less than half the number of close contacts than those under the age of 30 (52). Since US Asian older adults appeared more likely to socially isolate during COVID-19, and older adults in general had fewer close contacts, they might have been less likely to be exposed to everyday situations where they could experience discrimination.

Lower levels of perceived everyday discrimination during COVID-19 among Asian older adults might also be related to the specific characteristics of our sample. As shown in Table 1, Asian older adults were all foreign-born and reported their primary language as Mandarin or Cantonese. A prior study showed that US born Asian and Black individuals tend to report significantly more race-related discrimination than their foreign-born counterparts, suggesting that increased acculturation may shape the experience and perception of racial and ethnic discrimination (53).

Our results also indicate that average levels of depression and anxiety symptoms among Asian older adults remained stable during COVID-19 compared with their pre-pandemic levels. In addition, non-Asian older adults reported lower anxiety and depression symptoms during COVID-19 compared with before COVID-19. Older adults have experienced disproportionately greater adverse effects from the pandemic (54). However, data on the initial mental health effects of the pandemic on older adults present a much more nuanced picture. While some studies have shown that older adults' mental health deteriorated during the initial and later phases of the COVID-19 pandemic compared with before the pandemic (55, 56), our results are consistent with contrasting evidence suggesting a more limited negative effect on older adults' mental health (57, 58). A longitudinal prospective cohort study of older adults in the Netherlands, for example, showed that absolute changes in depression and anxiety symptoms during the pandemic compared with pre-pandemic were small and nonsignificant (57). In a sample of US older adults with pre-existing depression from the OPTIMUM clinical trial (a multisite comparative effectiveness trial of antidepressant treatments), depression and anxiety symptoms in the first 2 months of the pandemic were significantly lower than their baseline pre-pandemic and pre-treatment levels, indicating that participants did not relapse to pre-treatment levels of depression and anxiety at the beginning of the pandemic (58). In the present study, we used a sample of US older adults with pre-existing elevated depression or anxiety symptoms from the PMSB clinical trial. Our results suggest that older adults with pre-existing depression or anxiety might show resilience to the negative effects of the pandemic on mental health. This resilience to adverse mental health outcomes in late life has been suggested to reflect an interaction of internal factors (e.g., biological stress response, cognitive capacity, personality) and external resources (e.g., social status, financial stability) (54, 59). Indeed, prior evidence demonstrates that older adults tend to have lower stress reactivity and better emotional regulation and wellbeing than younger adults (54, 60).

We found that everyday discrimination was not associated with worse depression and anxiety symptoms, lower levels of functioning, or increased sleep difficulties pre-pandemic. Older adults from the present study were eligible for the PMSB trial based on pre-existing mental and physical conditions, and they were also not receiving mental health services. There is increasing evidence suggesting that people with mental illnesses who are not receiving care avoid treatment because of stigma and expected discrimination (61). Thus, the negative effects of perceived everyday discrimination could have already impacted the mental and physical health of older adults in our sample prior to the pandemic. In contrast, in the COVID-19 follow-up, we observed a significant positive correlation between self-reported discrimination and depression and anxiety symptoms and a negative correlation between discrimination and level of functioning, suggesting that the pandemic might have exacerbated the negative impact of discrimination on health. Our findings also suggested that this negative impact of everyday discrimination on health outcomes during COVID-19 appeared to affect Asian and non-Asian older adults similarly. That is, the effect of discrimination on health outcomes during COVID-19 was not stronger among Asian older adults compared to non-Asian older adults. Our results are consistent with recent studies demonstrating that racial discrimination has negatively impacted Asian populations during the pandemic (1, 2, 4), and they are also consistent with prior evidence demonstrating that racial discrimination negatively affects other racial and ethnic groups, including African American, Latinx, and White populations (3). Because discrimination did not appear to affect older adults' health before the pandemic, our results thus suggest that the pandemic might have made the effect of discrimination more salient to older adults overall (and not only Asian older adults).

A plausible explanation for why discrimination negatively impacted mental and physical health during but not pre-pandemic is that older adults may have had fewer resources to cope with discrimination during the pandemic. For example, a recent study showed that US Asian adults who experienced discrimination during COVID-19 appeared to use social media as a coping tool, and social media use was associated with better subjective wellbeing (62). Prior studies suggest that social media interactions with close contacts (such as private messaging and posting) might protect against the negative effect of discrimination because such interactions have a robust association with perceived social support (62, 63). However, US Asian older adults (aged 60 and above) who experienced discrimination during COVID-19 had the lowest levels of social media use (62). A recent survey also showed that over one-third of Asian American older adults in New York City did not have access to the internet during COVID-19, and over half were not comfortable using the internet even if they had access to it (13, 64).

Another plausible explanation is that the negative associations observed during the pandemic could be related to the specific type of discrimination measured in the present study. Since everyday discrimination assessed day-to-day experiences with unfair treatment, older adults who reported discrimination during COVID-19 are potentially more likely to have experienced such discrimination on a personal level (rather than indirectly through channels such as media, for example). A prior study found that observed discrimination through media during the COVID-19 pandemic was not a strong predictor of greater anxiety, vigilance, and worry (65). On the other hand, experiencing discrimination on a personal level during the pandemic was more traumatizing and intense (65).

Our last set of results indicated that social support and social cohesion offset the impact of everyday discrimination on depression symptoms and level of functioning during the COVID-19 pandemic, despite not being directly linked to better mental and physical outcomes. We found that although everyday discrimination during COVID-19 had a negative impact on depression symptoms and level of functioning for all older adults, the negative effect of discrimination on these two outcomes was attenuated at higher levels of social support and cohesion. This result supports the stress-buffering model, which posits that social support protects mental health through the indirect pathway of interacting with the stressor (i.e., discrimination) rather than by being directly associated with lower levels of psychological distress (28). According to our results, the stress-buffering effect occurred because social support and cohesion were not directly associated with lower psychological distress. Instead, higher levels of social support and cohesion appeared to lessen older adults' reaction to discrimination during the pandemic. The lack of direct association between social support and cohesion and health outcomes could also be explained by the fact that most older adults in our sample were foreign-born. A recent study on the association between social support and health outcomes among first-generation immigrant, second-generation immigrant, and non-immigrant US older adults found that while social support is strongly and positively associated with health in the general population, this association is null and in some cases even reversed among immigrants in the first and second generations (66). Nevertheless, our finding that social support and cohesion partially offset the impact of everyday discrimination is consistent with a sizable body of work showing that social support is a protective factor that buffers against the negative effect of discrimination on health, including during the COVID-19 pandemic (4, 30, 31). The protective effect of social support may be especially salient to Asian populations whose traditional cultural values of collectivism emphasize the importance of positive social relationships for their wellbeing (67).

## Limitations and conclusion

Our study has several limitations. The COVID-19 follow-up assessment started in March 2021, a year after the World Health Organization declared the COVID-19 outbreak a global pandemic. Thus, the effect of the COVID-19 pandemic on discrimination and mental and physical health at the early stages of the pandemic may differ from those we found in the present study. For example, a prior longitudinal study found that depression and anxiety symptoms sharply increased right at the beginning of the pandemic, but they rapidly declined within the next 20 weeks (68). Our study is thus limited in its ability to characterize full trajectories of discrimination and mental and physical outcomes before and during the pandemic. In addition, due to the small sample size, older adults were categorized into Asian and non-Asian groups. Future studies that include adequate numbers of racially and ethnically diverse older adults could shed light on the effect of discrimination on health outcomes during COVID-19 for other racial and ethnic groups, which may yield different results. While our study found that Asian and non-Asian groups reported lower perceived everyday discrimination during the COVID-19 follow-up, our study did not assess COVID-19 related discrimination specifically, but rather day-to-day experiences of unfair treatment. Response patterns might have been different if discrimination was assessed in relation to the COVID-19 pandemic, particularly among Asian older adults. In addition, internal consistency for our measures of sleep difficulties and social support were low (< 0.7). We did not find evidence that everyday discrimination was associated with increased sleep difficulties either before or during the pandemic. We also found that social support buffered against the negative effect of discrimination on depression symptoms during the pandemic, but it was not directly associated with either outcome. Given the low internal consistency, we cannot rule out the possibility that our results could have been different had these measures had better internal consistency. Lastly, our study sample was comprised of older adults previously enrolled in an RCT, and eligible based on elevated depression or anxiety symptoms and limited physical functioning, so our findings might not necessarily be applicable to the general population of US older adults. In addition, exclusion criteria included participants disclosing substance use disorders, but prior studies suggest that perceived racial discrimination is associated with increased risk of substance use (69, 70). Although only eight participants were excluded from the RCT because of substance use, future studies examining the association between discrimination and increased risk of substance use among older adults amid the COVID-19 pandemic are needed.

Notwithstanding these limitations, our study represents one of the first efforts to investigate within-person changes in day-to-day experiences of unfair treatment before and during the COVID-19 pandemic, and how these experiences of discrimination might have impacted health outcomes differently before vs. during the pandemic among Asian and non-Asian older adults. Our findings suggest that although older adults might have been less likely to be exposed to everyday situations where they could experience discrimination during COVID-19, the pandemic might still have exacerbated the negative impact of discrimination on health outcomes. However, social support and social cohesion can act as protective factors that buffer against this increased negative impact of discrimination. Our results suggest that public health interventions aimed at reducing everyday discrimination and emphasizing social support can potentially improve health outcomes of all US older adult populations, particularly those with pre-existing depression, anxiety, and functional limitations.

## Data availability statement

The data used to obtain the results presented in this manuscript is not publicly available because it involves a racial and ethnic minority sample including participants with depression, anxiety, and functional limitations. The author/s are not able to release the data used in the current manuscript given the sensitivity of the data and their agreements with the Institutional Review Boards of the participating institutions. Reasonable requests to access the data can be directed to Sheri Markle, at smarkle@mgh.harvard.edu.

## Ethics statement

Study procedures involving human participants were reviewed and approved by the Institutional Review Boards of Massachusetts General Hospital/Partners HealthCare and New York University, with ceded reviews for partnering Community-Based Organizations conducting human subjects research. The patients/participants provided their written informed consent to participate in this study.

## Author contributions

LZ, MC-G, and MA contributed to the conception, design of the study, and wrote the first draft of the manuscript. LZ and MC-G organized the database and performed the statistical analysis. ZL, XO, and FZ wrote sections of the manuscript. All authors contributed to manuscript revision and approved the submitted version.

## Funding

Research reported in this publication was supported by the National Institute on Aging and the National Institute of Mental Health under Grant Number R01AG046149 and its COVID supplement. The funders (NIA, NIMH) had no role in design and conduct of the study; collection, management, analysis, and interpretation of the data; preparation, review, or approval of the manuscript; or decision to submit the manuscript for publication.

## Conflict of interest

The authors declare that the research was conducted in the absence of any commercial or financial relationships that could be construed as a potential conflict of interest.

## Publisher's note

All claims expressed in this article are solely those of the authors and do not necessarily represent those of their affiliated organizations, or those of the publisher, the editors and the reviewers. Any product that may be evaluated in this article, or claim that may be made by its manufacturer, is not guaranteed or endorsed by the publisher.

## Author disclaimer

The content is solely the responsibility of the authors and does not necessarily represent the official views of the National Institutes of Health.
